# Lower serum uric acid levels as a risk factor for depression in prodromal Parkinson’s disease: a cohort study

**DOI:** 10.1515/biol-2025-1309

**Published:** 2026-04-20

**Authors:** Keke Liang, Yetong Ouyang, Bingyu Li, Tao Li, Jun Tan, Chenxi Shuai, Xiaohui Tang, Zhilin Chen, Zhexue Huang, Xiaoshun Tang, Jiayi Jin, Qian Liang, Zhengchen Li, Qing-Ran Bai, Xijin Wang

**Affiliations:** Department of Neurology, Shanghai Tongji Hospital, School of Medicine, Tongji University, Shanghai 200065, China; Key Laboratory of Spine and Spinal Cord Injury Repair and Regeneration of Ministry of Education, Department of Orthopedics, Tongji Hospital, School of Medicine, Tongji University, Shanghai 200092, China

**Keywords:** prodromal Parkinson’s disease, depression, serum uric acid, risk factor

## Abstract

Depression, as an important prodromal non-motor manifestation of Parkinson’s disease (PD), its early predictive indicators remain unclear. Although cross-sectional studies suggest an association between serum uric acid (UA) levels and depression in PD, longitudinal evidence in the prodromal period is lacking. This study innovatively conducted a 5-year longitudinal study in a population with prodromal Parkinson’s disease (pPD), revealing for the first time the longitudinal association between serum UA levels and the development of depression. At baseline, we selected 460 pPD patients from the Parkinson’s Progression Markers Initiative (PPMI) database. Eventually, 61 participants completed a 5-year longitudinal study with annual assessments. Predictor variables were selected through univariate Cox analysis and LASSO regression, with multivariate Cox regression models employed to assess the independent association between serum UA levels and incident depression in the pPD cohort. At baseline, 18.9 % of pPD patients developed depression, and the cumulative incidence rates of depression during the 5-year follow-up period were 34.43 %, 49.18 %, 57.38 %, 62.30 % and 63.93 %, respectively. Multivariate Cox regression analysis, adjusted for covariates including sex and age, identified a higher Movement Disorder Society Unified Parkinson’s Disease Rating Scale Part I (MDS-UPDRS I) score (HR = 1.177, 95 % CI 1.093–1.267, *P* < 0.001) and lower serum UA levels (HR = 0.776, 95 % CI 0.619–0.974, *P* = 0.029) as independent risk factors for incident depression in the pPD cohort. Our study shows that lower serum UA levels can independently predict the risk of depression in pPD, providing a new biomarker for the early identification of high-risk populations.

## 1 Introduction

Parkinson’s disease (PD), the second most prevalent neurodegenerative disorder globally, is projected to double in prevalence over the next three decades [1]. The clinical manifestations mainly include motor symptoms such as bradykinesia, resting tremor and rigidity, as well as various non motor symptoms [2], 3]. The onset of PD is insidious, and the progression of the disease is slow, there may be a long prodromal period before the clinical symptoms of PD [4], 5]. Depression, as one of the common non-motor symptoms in PD patients, has been extensively studied [6], 7]. Notably, depressive symptoms frequently manifest during the prodromal stage, predating motor deficits, and are associated with accelerated disease progression, reduced quality of life, and elevated risks of disability and mortality [8], 9]. However, the relationship between depression and biomarkers associated with prodromal Parkinson’s disease (pPD) has not been fully elucidated.

Uric acid (UA), an extracellular antioxidant synthesized exclusively from purine metabolism, has been implicated in the pathogenesis and progression of neurodegenerative disorders, including Alzheimer’s disease and multiple sclerosis [10], 11]. Cross-sectional studies have indicated an association between serum UA and depression in PD [12], [13], [14], [15], and there is a lack of cohort studies. This study represents the first to include participants with pPD in a 5-year longitudinal investigation, aiming to explore whether dynamic UA fluctuations correlate with the temporal trajectories of depressive symptoms, with a view to providing new evidence for the early identification and intervention of depression in pPD patients.

## 2 Materials and methods

### 2.1 Research samples

The data used in this study were obtained from Parkinson’s Progression Markers Initiative (PPMI) database (www.ppmi-info.org/data). PPMI is an international, observational, cohort study with almost 50 sites across 13 countries. Each site participating in the PPMI was approved by the Ethics Standards Committee for Human Experimentation before the start of the study, and at each site, all subjects signed an informed consent form [16]. As this retrospective analysis exclusively utilized anonymized, publicly available datasets posing no physical risks to participants, the Ethics Committee of Tongji University School of Medicine Shanghai Tongji Hospital granted an exemption from additional ethical review.

**Informed consent:** Informed consent has been obtained from all individuals included in this study.

**Ethical approval:** The research related to human use has been complied with all the relevant national regulations, institutional policies and in accordance with the tenets of the Helsinki Declaration, and has been approved by the authors’ Institutional Review Board or equivalent committee.

### 2.2 Object selection

**Figure 1:**
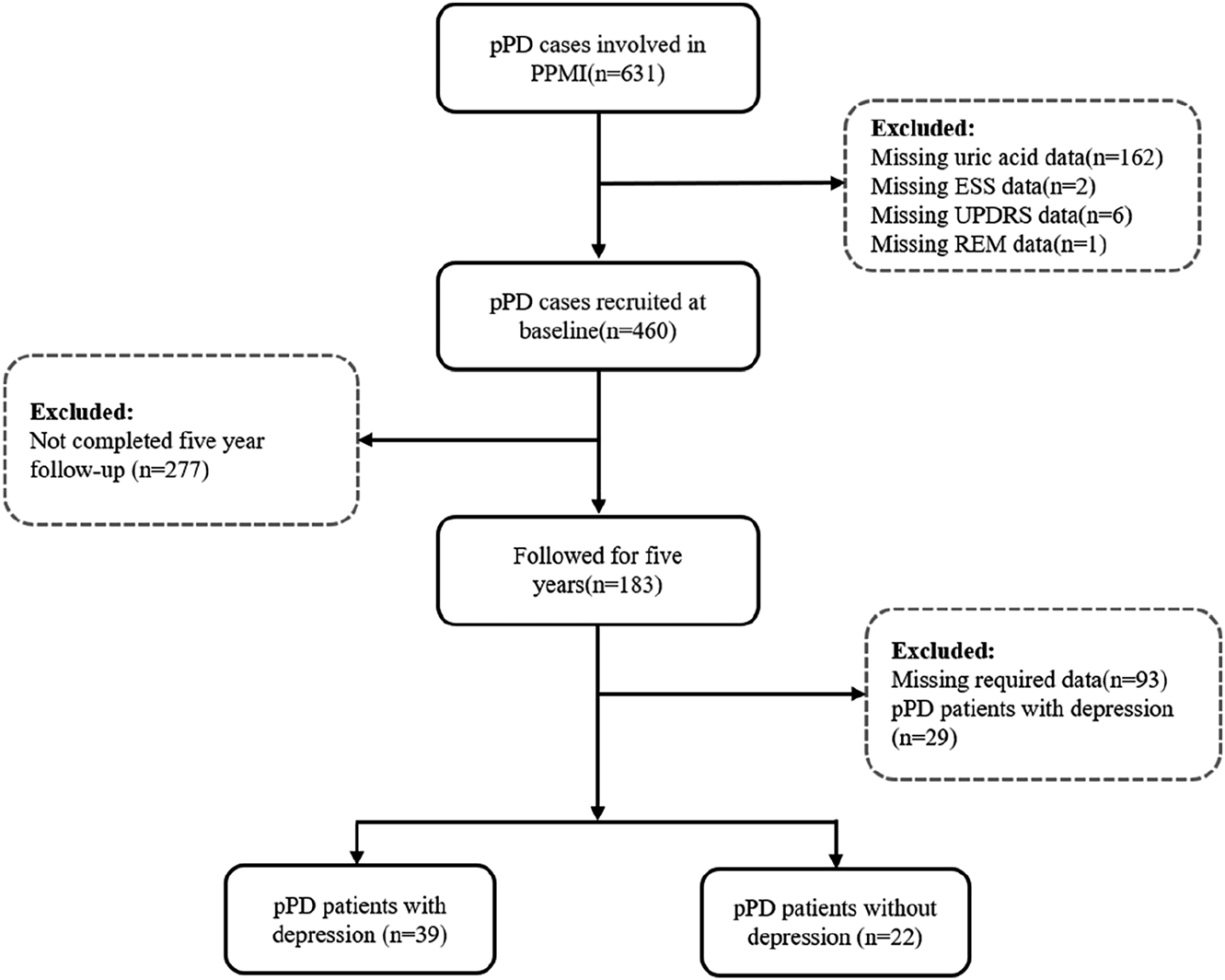
Flow chart of the selection of research objects.

This prospective cohort study analyzed individuals with pPD, defined as exhibiting early neurodegenerative manifestations of PD without meeting formal motor diagnostic criteria [17]. Details of the pPD inclusion/exclusion criteria adopted by the PPMI can be found at http://www.ppmi-info.org [16], 18]. Data used in the preparation of this article were obtained on June 12th, 2023 from the PPMI database (www.ppmi-info.org/access-data-specimens/download-data, RRID:SCR_006431). For up-to-date information on the study, visit www.ppmi-info.org. From an initial pool of 631 participants, 460 met baseline eligibility after excluding cases with incomplete data. Using Movement Disorder Society Unified Parkinson’s Disease Rating Scale (MDS-UPDRS) item 1.3, the cohort was stratified into depression (*n* = 87) and non-depression (*n* = 373) subgroups. A subset of 183 participants underwent annual assessments over five years. In pPD patients, the occurrence of depression was used as the outcome variable, and the date on which depression first appeared during the follow-up period without depression at baseline was recorded. After excluding baseline depression cases and incomplete datasets, 61 pPD participants comprised the final analytical cohort, as shown in Figure 1.

### 2.3 Clinical characteristics and evaluation

The MDS-UPDRS assessed motor and non-motor symptoms [19], with items 1.3 and 1.5 specifically evaluating depression and apathy in pPD participants. Disease severity was classified using the Hoehn-Yahr (H–Y) stage. Cognitive function was measured via the Montreal Cognitive Assessment [20], which has a total score of 30 points, the lower the score, the lower the cognitive function. Excessive daytime sleepiness (EDS) was defined as Epworth Sleepiness Scale (ESS) scores ≥10 [21], 22]. Anxiety levels were quantified using the State–Trait Anxiety Inventory (STAI), with higher scores reflecting greater anxiety [23]. Autonomic dysfunction was evaluated with the Scales for Outcomes in Parkinson’s Disease-Autonomic (SCOPA-AUT) questionnaire [24]. Both motor and nonmotor symptom scores were assessed during the “On” period.

### 2.4 Biomarker determination

The available biofluid biomarkers included cerebrospinal (CSF) Aβ1-42, CSF total tau (t-tau), CSF phosphorylated tau (p-tau), serum UA and serum neurofilament light (NfL). For detailed information on biological sample collection and processing, please refer to http://www.ppmi-info.org [25].

### 2.5 Statistical analysis

GraphPad Prism version 8.0 (GraphPad Software, Inc., San Diego, CA, USA.) software was used for graphing, and R version 4.2.3 (R Foundation for Statistical Computing, Vienna, Austria) software was used for statistical analysis. Normally distributed continuous variables are reported as mean ± standard deviation (SD) and compared using independent two-sample *t*-tests. Non-normally distributed variables are expressed as median [*M* (*Q1, Q3*)] and analyzed via Mann–Whitney U tests. Categorical variables were compared with Chi-square tests and Fisher’s exact tests. Univariate Cox and LASSO regression analysis were used for feature variable screening, and multivariate Cox regression was used to assess independent risk factors for depression in pPD patients, with statistical significance defined as *P* < 0.05.

## 3 Results

### 3.1 Clinical and demographic characteristics of pPD patients at baseline

This study enrolled 631 individuals with pPD, of whom 460 were included in the baseline analysis. Table 1 summarizes the demographic and clinical characteristics of pPD patients with depression (*n* = 87) and without depression (*n* = 373). In our study, the difference between the groups were statistically significant only in sex (*P* = 0.027), MDS-UPDRS I score (*P* < 0.001), MDS-UPDRS total score (*P* < 0.001), apathy (*P* < 0.001), STAI total score (*P* < 0.001) and SCOPA-AUT total score (*P* = 0.019).

**Table 1:** Clinical and demographic characteristics of pPD patients.

Variable	pPD without depression (*n* = 373)	pPD with depression (*n* = 87)	*P* value
Age (years)	63.09 (59.17–68.50)	63.55 (57.54–71.51)	0.811
Sex (male/female)	182/191	31/56	0.027
BMI (kg/m^2^)	27.00 (24.00–30.75)	25.00 (22.75–30.00)	0.355
Education (years)	18.00 (16.00–19.00)	16.00 (13.00–18.25)	0.369
LEDD	0 (0–0)	0 (0–0)	0.734
MDS-UPDRS I score	4.00 (2.00–6.75)	8.0 (4.75–11.25)	<0.001
MDS-UPDRS II score	0 (0–2)	0 (0–2)	0.567
MDS-UPDRS III score	1.00 (0–4.00)	3 (1.00–4.50)	0.114
MDS-UPDRS total score	7.00 (3.00–12.75)	13.00 (8.00–15.25)	<0.001
H–Y	0 (0–0)	0 (0–0)	0.057
MoCA score	27.00 (25.00–28.00)	27.00 (25.75–28.00)	0.430
EDS [*n* (%)]			0.127
≥10	42 (11.26)	15 (17.24)	
<10	331 (88.74)	72 (82.76)	
Apathy [*n* (%)]			<0.001
Yes	18 (4.83)	32 (36.78)	
No	355 (95.17)	55 (63.22)	
STAI total score	54.00 (45.00–66.00)	72.50 (65.25–92.50)	<0.001
SCOPA-AUT total score	7.00 (5.00–11.75)	10.00 (7.00–17.00)	0.019
CSF Aβ1-42 (pg/mL)	867.50 (674.25–1,040.75)	991.00 (757.00–1,162.00)	0.191
CSF t-tau (pg/mL)	159.00 (129.75–199.75)	171.00 (130.75–215.00)	0.258
CSF p-tau (pg/mL)	14.00 (11.00–17.00)	14.50 (11.75–18.25)	0.337
Serum NfL (pg/mL)	12.50 (10.00–17.00)	15.00 (9.75–19.25)	0.314
Serum UA (μmol/L)	356.88 (237.92–356.88)	297.40 (279.56–356.88)	0.844

BMI, body mass index; MoCA, montreal cognitive assessment; CSF, cerebrospinal fluid.

### 3.2 Cumulative incidence at 5-year follow-up in pPD patients with depression

**Figure 2:**
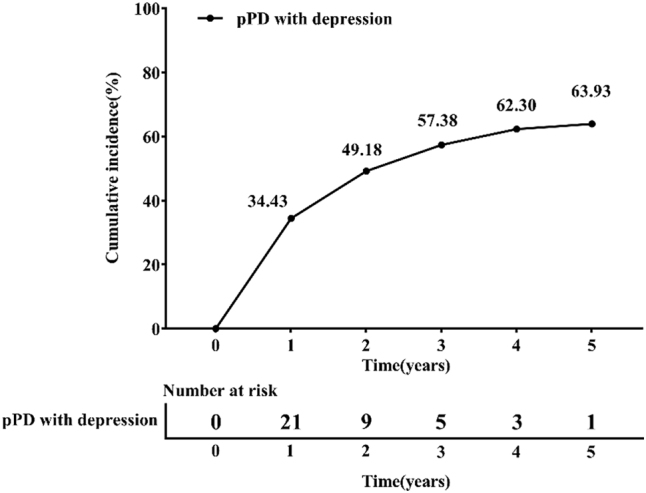
Cumulative annual incidence of depression in pPD patients.

Among 61 pPD participants who completed the five-year follow-up, 39 developed depressive symptoms during the observation period. Figure 2 presents the cumulative incidence of depression in the pPD cohort over this duration, with rates progressively increasing to 34.43 % at year 1, 49.18 % at year 2, 57.38 % at year 3, 62.30 % at year 4, and 63.93 % at year 5.

### 3.3 Predictors of the occurrence of depression in pPD patients

**Figure 3:**
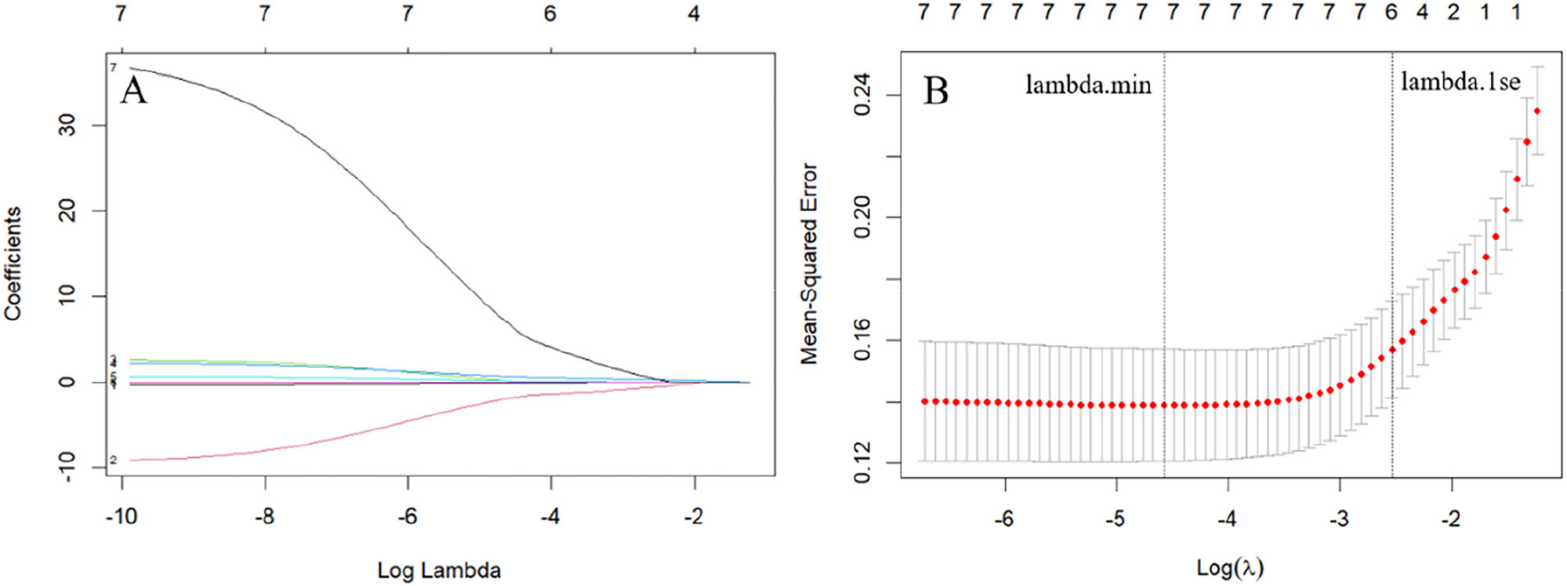
Identification of key risk factors for depression in prodromal Parkinson’s disease using LASSO regression analysis. (A) LASSO coefficient profiles of the eight risk factors. This study included eight variables, resulting in eight lines of different colors. Each colored trace represents the regularization path of a predictor variable, with the *y*-axis indicating coefficient magnitudes, the lower *x*-axis displaying log(λ) penalty values, and the upper *x*-axis showing the count of non-zero coefficients retained in the model. (B) Three risk factors selected using LASSO regression analysis. The *X*-axis is the logarithmic log *λ* of the penalty coefficient, and the *Y*-axis is the likelihood deviation. The smaller the *Y*-axis, the better the fitting effect of the equation. The top axis denotes the number of retained predictors at corresponding *λ* thresholds. Two vertical dashed lines mark critical *λ* values: the minimum mean squared error and the one-standard-error rule, optimizing the balance between model complexity and predictive accuracy. Color coding in panel A corresponds to individual predictor variables as listed in Table 2.

**Figure 4:**
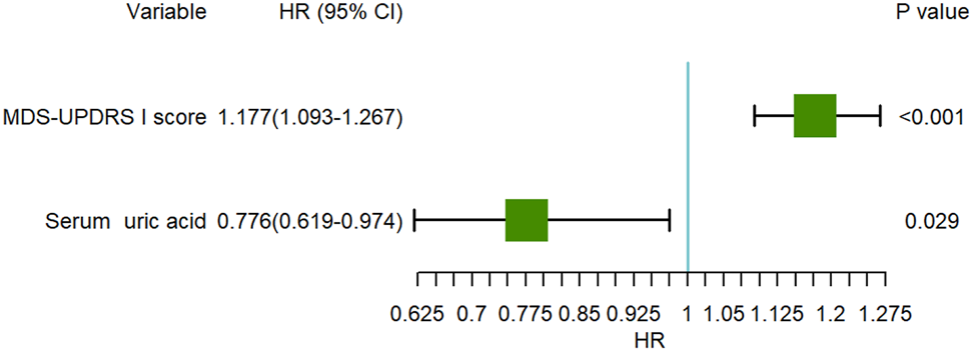
Forest plot of multivariable COX regression analysis.

Using the pPD patients with depression as a reference, univariate Cox regression model was applied to show that the following variables are associated with pPD patients with depression: age, sex, Education, MDS-UPDRS I score, STAI, apathy and serum UA. To mitigate collinearity and overfitting while enhancing model stability and predictive accuracy, LASSO regression was employed for feature selection. Variables with univariate *P* ≤ 0.1 were subjected to LASSO regression, revealing significant associations with depression for younger age (*β* = −0.009), female (*β* = −0.169), higher education (*β* = 0.001), higher MDS-UPDRS I score (*β* = 0.047), higher STAI (*β* = 0.004), higher apathy (*β* = 0.227) and lower serum UA (*β* = −0.014). Seven variables retained by LASSO regression were subsequently analyzed via multivariate Cox regression (Figure 3). After adjustment for covariates including sex and age, the analysis revealed that higher MDS-UPDRS I scores (HR = 1.177, 95 % CI 1.093–1.267, *P* < 0.001) and lower serum UA (HR = 0.776, 95 % CI 0.619–0.974, *P* = 0.029) independently predicted depression in pPD (Table 2, Figure 4). The combined model demonstrated adequate goodness of fit (*χ* ^2^ = 22.152; *df* = 1; *P* < 0.001).

**Table 2:** Cox regression and Lasso regression for the occurrence of depression in pPD patients.

Variable	Univariable analysis		Lasso regression analysis	Multivariable analysis	
	HR (95 % CI)	*P* value	*β*	HR (95 % CI)	*P* value
Age (years)	0.965 (0.933–0.997)	0.034	−0.009	NA	NA
Sex	0.427 (0.220–0.830)	0.012	−0.169	NA	NA
BMI (kg/m^2^)	0.943 (0.885–1.005)	0.072	0	NA	NA
Education (years)	1.114 (1.021–1.215)	0.015	0.011	NA	NA
LEDD	0.999 (0.996–1.002)	0.452	NA	NA	NA
H–Y	1.093 (0.705–1.695)	0.690	NA	NA	NA
MDS-UPDRS I score	1.162 (1.085–1.245)	<0.001	0.047	1.177 (1.093–1.267)	<0.001
MDS-UPDRS II score	0.993 (0.916–1.074)	0.864	NA	NA	NA
MDS-UPDRS III score	0.991 (0.947–1.038)	0.703	NA	NA	NA
MDS-UPDRS total score	1.013 (0.989–1.037)	0.308	NA	NA	NA
MoCA score	1.024 (0.905–1.158)	0.707	NA	NA	NA
EDS	1.098 (0.504–2.393)	0.815	NA	NA	NA
STAI	1.024 (1.009–1.040)	0.002	0.004	NA	NA
Apathy	2.302 (1.049–5.056)	0.038	0.227	NA	NA
SCOPA-AUT total score	1.001 (0.961–1.043)	0.954	NA	NA	NA
CSF Aβ1-42 (pg/mL)	1.000 (0.999–1.002)	0.771	NA	NA	NA
CSF t-tau (pg/mL)	0.998 (0.993–1.004)	0.554	NA	NA	NA
CSF p-tau (pg/mL)	0.974 (0.915–1.037)	0.414	NA	NA	NA
Serum NfL (pg/mL)	0.991 (0.937–1.047)	0.736	NA	NA	NA
Serum UA (μmol/L)	0.796 (0.639–0.993)	0.043	−0.014	0.776 (0.619–0.974)	0.029

### 3.4 Sensitivity analysis

In this study, the primary analysis was based on data from 61 patients who completed the 5-year follow-up. To effectively mitigate the potential impact of selection bias on the study results, we meticulously employed the scientific strategy of comparing baseline characteristics. The baseline population was divided into the group included in the Cox regression analysis and the group excluded due to no follow-up or insufficient follow-up for a systematic comparison of demographics, clinical measures, and biomarkers to assess baseline comparability. The results showed that only depression-related measures were significantly different between the two groups, with no statistical differences in the remaining baseline indicators (Table 3).

**Table 3:** Baseline characteristics comparison between Cox regression-included group and lost-to-follow-up/inadequate follow-up excluded group.

Variable	Lost-to-follow-up/inadequate follow-up excluded group (*n* = 399)	Cox regression-included group (*n* = 61)	*P* Value
Age (years)	62.47 ± 7.77	64.32 ± 7.84	0.085
Sex (male/female)	184/215	29/32	0.835
BMI	27.00 (24.00–30.50)	26.00 (23.50–30.00)	0.734
Education (years)	18.00 (15.50–19.00)	17.00 (14.00–19.00)	0.190
LEDD	0 (0–0)	0 (0–0)	0.844
MDS-UPDRS I score	4.00 (2.00–7.50)	4.0 (2.00–8.50)	0.353
MDS-UPDRS II score	0 (0–2)	1 (0–2)	0.430
MDS-UPDRS III score	1.00 (0–4.00)	1.00 (0–5.00)	0.336
MDS-UPDRS total score	7.00 (4.00–13.00)	8.00 (4.00–14.00)	0.425
H–Y	0 (0–0)	0 (0–0)	0.932
MoCA score	27.00 (26.00–28.00)	27.00 (24.00–28.00)	0.677
EDS [*n* (%)]			0.064
≥10	45 (11.30)	12 (19.70)	
<10	354 (88.70)	49 (80.30)	
Apathy [*n* (%)]			0.471
Yes	45 (91.80)	5 (8.20)	
No	354 (88.70)	56 (91.80)	
Depression			<0.001
Yes	87 (21.80)	0 (0)	
No	312 (78.20)	61 (100)	
STAI total score	57.00 (47.50–69.00)	62.00 (46.00–70.50)	0.917
SCOPA-AUT total score	7.00 (5.00–12.00)	8.00 (5.00–16.00)	0.265
CSF Aβ1-42 (pg/mL)	684.50 (869.00–1,076.00)	872.00 (706.00–1,020.50)	0.658
CSF t-tau (pg/mL)	160.00 (123.00–201.50)	161.00 (137.00–205.50)	0.542
CSF p-tau (pg/mL)	14.00 (11.00–17.00)	14.00 (11.00–18.00)	0.785
Serum NfL (pg/mL)	13.00 (9.00–17.00)	14.00 (11.00–17.50)	0.648
Serum UA (μmol/L)	356.88 (297.40–356.88)	297.40 (237.92–356.88)	0.324

## 4 Discussion

In this cohort study, we conducted a 5-year longitudinal assessment of depressive symptoms in pPD patients. The results revealed a progressive increase in the cumulative incidence of depression, rising from 18.9 % at baseline to 63.93 % by the study endpoint. These findings indicate that pPD patients face a heightened risk of developing depression as the disease advances, with the likelihood of depressive episodes escalating over time. This underscores the necessity for sustained monitoring and targeted interventions in this population. Following multivariate Cox regression analysis adjusted for potential confounders including gender and age, elevated MDS-UPDRS Part I scores and reduced serum UA levels emerged as independent risk factors for depression in pPD patients. This discovery provides critical insights into the pathophysiological mechanisms underlying depression in pPD and lends empirical support to the hypothesized neuroprotective role of UA.

Serum UA levels, as the primary variable of interest in this study, hold significant implications for understanding depression in PD. To date, most investigations exploring the relationship between serum UA levels and PD-associated depression have employed cross-sectional designs [26], 27], which can reveal the correlation between the two at a specific point in time, but cannot effectively elucidate the dynamic process of the disease. Notably, systematic cohort studies examining this relationship – particularly within the pPD subpopulation – remain absent in the literature. Our findings demonstrate that reduced serum UA levels are associated with an elevated risk of depression in pPD patients. There may be complex biological mechanisms underlying the association between serum UA levels and depression in pPD patients. As a potent antioxidant, UA scavenges reactive nitrogen and oxygen species, mitigates oxidative stress, and thereby exerts neuroprotective effects in PD [28], [29], [30], [31]. These protective properties have been validated at both cellular and animal model levels [32], 33]. Oxidative stress plays a key role in the pathogenesis of neurodegenerative diseases, excessive oxidative damage may drive neuronal apoptosis and disrupt neurotransmitter synthesis and metabolism [34], [35], [36]. Beyond its well-established antioxidant properties, UA may also modulate neuroinflammation, influence monoamine neurotransmitter transmission, and affect brain energy metabolism. Growing evidence suggests that UA can inhibit inflammasome activation and reduce the release of pro-inflammatory cytokines, thereby mitigating neuroinflammatory processes implicated in PD pathogenesis [28], 37]. Additionally, UA may interact with purinergic signaling pathways that regulate synaptic plasticity, neuroglial communication, and emotional processing [38], 39]. These multifaceted mechanisms may collectively contribute to UA’s neuroprotective effects and its association with depression in pPD. It should be noted that UA levels can be influenced by multiple factors, including renal function, genetic variations, medication use, comorbidities, and complex metabolic profiles. Despite the rich clinical features provided by the PPMI database, the current study, limited by data availability, could not comprehensively include all the aforementioned variables. Future research should aim to integrate these factors to more precisely elucidate the role of UA in the pathophysiology of neuropsychiatric disorders and its complex association with depression in pPD.

Diminished serum UA levels compromise the body’s antioxidant defenses, heightening neuronal vulnerability to oxidative stress, which may precipitate or intensify depressive symptomatology. This conclusion is consistent with previous studies on the relationship between neurological disorders and UA [11], further supporting the important role of UA in neuroprotection. Furthermore, emerging evidence implicates dysregulation of the central serotonergic system and neurodegenerative alterations within cortical-subcortical pathways in PD-associated depression [40], 41]. Purine-mediated signaling regulates neural transmission and may contribute to emotional pathophysiology by modulating neurotransmitter systems and hypothalamic-pituitary-adrenal axis activity [39]. As the terminal product of purine metabolism, UA influences the bioavailability of neurotransmitters such as dopamine and serotonin, both pivotal to affective regulation. Consequently, reduced serum UA levels may disrupt neurotransmitter equilibrium, thereby elevating depression risk.

The MDS-UPDRS I score quantifies non-motor symptom severity in PD, with elevated scores reflecting more pronounced impairments in mental, emotional, and behavioral domains. In this study, higher MDS-UPDRS I scores correlated with increased depression risk, potentially attributable to bidirectional interactions between non-motor symptom clusters [42]. Furthermore, elevated scores may reflect the severity of the disease, and patients with more severe conditions are more likely to experience psychological problems, such as depression. These findings underscore the imperative for clinicians to extend surveillance beyond motor symptom management in pPD patients and prioritize routine assessment and longitudinal monitoring of neuropsychiatric status. Early detection and intervention targeting non-motor symptoms could mitigate depression risk in this population.

This study offers methodological strengths by addressing a critical knowledge gap in understanding the relationship between serum UA levels and depression among patients with pPD. Through a 5-year longitudinal cohort design, it provides unique insights into depression trajectory monitoring within this population. However, this study also has some limitations. First, while adjustments were made for demographic confounders (e.g., age, sex), residual confounding from unmeasured variables (e.g., genetic predisposition, lifestyle habits) may influence observed associations. Second, the 5-year follow-up duration may preclude detection of delayed risk modifiers. Third, the high attrition rate (460 baseline participants, with only 61 completing the 5-year follow-up) may have introduced selection bias. Although we applied stringent inclusion criteria and excluded incomplete datasets, the potential impact of attrition on the generalizability of the findings cannot be ruled out. Therefore, we conducted a sensitivity analysis by comparing baseline characteristics, which indicated that attrition had minimal interference with the core conclusions, the risk of bias is manageable, and the findings remain robust. Future studies with larger sample sizes and longer follow-up durations are warranted to validate these observations. The conclusions of this study should be considered exploratory, and future research with larger sample sizes and extended follow-up durations is necessary to validate these observations.

## 5 Conclusions

This study confirms that lower serum UA levels and higher MDS-UPDRS I scores are independent risk factors for depression in pPD patients. These findings provide critical insights for clinicians to refine early detection and therapeutic strategies targeting serum UA modulation, which can help improve the mental health and quality of life of pPD patients.
